# Simulated Body Fluid-Assisted Stress Corrosion Cracking of a Rapidly Solidified Magnesium Alloy RS66

**DOI:** 10.3390/ma17163967

**Published:** 2024-08-09

**Authors:** R. K. Singh Raman, Lokesh Choudhary, Dan Shechtman

**Affiliations:** 1 Department of Mechanical & Aerospace Engineering, Monash University, Clayton, VIC 3800, Australia; lokesh.gdu@gmail.com; 2 Department of Chemical & Biological Engineering, Monash University, Clayton, VIC 3800, Australia; 3 Department of Materials Engineering, Technion-Israel Institute of Technology, Haifa 32000, Israel; danny.shechtman@gmail.com

**Keywords:** biodegradable implants, magnesium alloy, stress corrosion cracking, rapid solidification

## Abstract

This study investigated the simulated body fluid-assisted stress corrosion cracking (SCC) of an Al-free magnesium alloy (RS66) and a common Al-containing magnesium alloy (AZ91), the former being more suitable for temporary implant applications (however, we used AZ91 for comparison since there are considerable reports on SCC in this alloy). The investigation includes SCC tests under simultaneous conditions of mechanical loading and imposed electrochemical potential that established a combined effect of hydrogen and anodic dissolution as the embrittlement mechanism. Though the RS66 alloy possesses impressive mechanical properties in non-corrosive environments (as a result of its fine grain size), both alloys suffered significant embrittlement when tested in simulated body fluid. The susceptibility of the RS66 alloy to SCC was ~25% greater than that of AZ91, which is attributed to the greater resistance of AZ91 to corrosion/localised corrosion because of its Al content.

## 1. Introduction

It is highly attractive to construct temporary implants (such as pins, wires, screws, plates, stents) out of suitable magnesium alloys that may dissolve away in human physiological fluid after performing its function of holding the fractured bone together until complete re-joining/healing. The use of implants of such alloys that harmlessly and completely biodegrade/dissolve away after their function enables avoiding the burden of the second surgery, that is invariably required to remove the temporary implants constructed from the traditional materials (e.g., titanium alloys and stainless steels). Therefore, biocompatible Mg alloys have attracted forefront research attention [1,2]. Other attractive features of Mg alloys in their use as bioimplants are the following [3,4,5,6,7,8]:

(a) the mechanical properties of Mg alloys and human bone are close [3,4] (such as alloy density of 1.74–2.0 g cm^−3^ vis-à-vis 1.8–2.1 g cm^−3^ for bone, and alloy elastic modulus of 41–45 GPa vis-à-vis 3–20 GPa for bone), and

(b) Mg is biocompatible; Mg biodegrades into physiologically non-toxic products, is essential to the human metabolism [5,6] and assists in tissue healing and growth [7], and excess Mg^2+^ is excreted through renal system without causing any harm [8].

Therefore, Mg alloys (that have non-toxic alloying contents) with the required combination of the mechanical properties and bio-compatibilities are attractive for their application as temporary bioimplants.

Metallic materials in human implant applications can be susceptible to sudden and premature fracture/cracking due to the synergistic role of mechanical loading during human actions, and the body fluid, which is corrosive. The principal corrosion-assisted fracture modes are stress corrosion cracking (SCC), which requires tensile loading, and corrosion fatigue (CF) that requires cyclic loading. The aspects of SCC and CF fractures that cause concern are as follows: (a) even the stresses considerably below design stresses for the immune environment can be sufficient to cause SCC and CF, and (b) the alloy loses ductility (suffers embrittlement) due to SCC and CF that lead to premature brittle fracture. Such premature fractures often necessitate cumbersome removal, and surrounding tissues suffer irritation/inflammation. Further, the sharp contours of a device such as those in implants (e.g., screws) act as common sites for initiation of SCC and CF cracks, as seen in the case of implants of common alloys (i.e., stainless steels, Ti-alloys, and Co-Cr alloy [9,10,11]). SCC is likely to be a considerable concern for Mg alloys in their application as implants since:

(a) magnesium alloys readily suffer pitting in aqueous chloride environments [12,13], including in the human physiological environment (and, as described earlier, CF and SCC often initiate from sharp contours such as pits), and

(b) contours of most temporary implants, e.g., pins, screws, and plates are sharp.

Indeed, there are reported observations of SCC in Mg alloys in chloride solutions [14,15]. However, there are very limited studies on simulated body fluid-assisted SCC of Mg alloys [15,16,17,18,19,20,21,22]. Before actual use of magnesium alloys as biodegradable implants, comprehensive studies are required on the SCC and CF of such alloys [23].

For their use as bioimplants, it is necessary to select such magnesium alloys that have alloying constituents that are required for mechanical strength and resistance to corrosion in human body fluid, without causing any toxicity [23,24,25,26,27,28,29,30], as well as having the required resistance to SCC or CF. Al, Zn, Ca, and rare earths (REs) are among the alloying elements used in Mg alloys for the purpose of mechanical strength and corrosion resistance. Since aluminium (Al) can confer both strength and corrosion resistance, Al-containing Mg alloys (i.e., AZ series alloys) are popular. But, Al is generally believed to be toxic as it can cause Alzheimer disease and dementia [24], thus ruling out the use of the AZ-series Mg alloys for biodegradable implants (note, the implant alloy will be required to dissolve away within the human body after fulfilling the temporary function). Ca is a major constituent of bones, and the surface of a Ca-containing magnesium alloy rapidly develops a hydroxy apatite layer, which facilitates biocompatibility in the human body [31]. Ca is also essential for human physiology such as chemical signalling in the cyto-system [20]. In addition, Ca addition to Mg alloys also facilitates grain refinement, which strengthens the alloys [23]. However, Mg_2_Ca precipitates that form along alloy grain boundaries at Ca contents > 1 wt.% can embrittle the alloy [31] and compromise its mechanical properties. Alloying with Zn has a solid solution-strengthening effect. But, excessive Zn addition (≥6.2 wt.%) too can cause embrittlement (due to Mg-Zn precipitation). Because of the beneficial effects of both Zn and Ca additions which help meet the non-toxicity criteria, Mg-Zn-, Mg-Ca-, and Mg-Zn-Ca-type alloys [1,6,31,32] have been studied for implant applications. Rare earths (REs), when added in sufficient quantities to Mg-alloys, have been reported to considerably improve the alloys’ corrosion resistance [32,33]. However, the reports on toxicity due to REs are generally inconclusive/insufficient [4,7].

The alloying contents in Mg alloys are required to be selective, on the basis of their profound role in corrosion resistance, non-toxicity and strengthening, and importantly, the embrittlement of the alloys. As described earlier, an excessive alloying content can cause intermetallic precipitations that can embrittle the alloy. The embrittling influence will be more pronounced in a corrosive environment, as a result of the profound role of intermetallic phases (that are highly cathodic) in triggering localized corrosion/pitting [12,13] and SCC, since the pits are the common crack initiators. However, alloys with insufficient alloying contents will fail to provide the desired corrosion resistance. In attempts to circumvent this challenge, magnesium alloys with lean alloying contents have been developed [34,35]. Another approach is to rapidly solidify the magnesium alloys with sufficient alloying contents, which results in grain refinement and suppression of intermetallic precipitation [1,2]; the latter can also suppress susceptibility to SCC [20]. Such approaches have led to some remarkable advancements in the application of magnesium alloys as bioimplants [1,2,34,35,36,37]. Mg-Zn-Y-Ce-Zr alloy, RS66, an alloy that was developed upon rapid solidification and reciprocal extrusion by the co-author Shechtman and colleagues [38], possesses an excellent combination of properties, viz., high strength and ductility, and homogeneous distribution of grain size (~1 μm). In-vivo and in-vitro testing has established the RS66 alloy as also meeting the requirements of biodegradation and biocompatibility (with no toxicity effects) for bioimplant applications [39]. Biocompatibility tests of RS66 have shown no clinical harm in the 6 weeks in subcutaneous and intramuscular implantation sites [39]. Since RS66 corrodes at low rates, which is similar to a few earlier rapidly solidified alloys [1,2], the alloy also possesses the important characteristic of not generating ‘clinically observable hydrogen’ levels [39], which has been one of the problems in the use of magnesium alloys as bioimplants.

There is very little reported on the simulated body fluid (SBF)-assisted SCC of rapidly solidified and extruded Mg alloys [20]. The majority of studies on SCC and CF of Mg alloys in SBF are limited to AZ series alloys, such as AZ91 [16,17,21,22], which contain toxic aluminium. Though these alloys cannot be used as implants, the studies have confirmed the investigated alloys to suffer SCC in SBF (as both mechanical data and fractography confirmed expedited fracture in SBF). The reported studies also enabled a mechanistic understanding of the SCC of magnesium alloys in SBF (i.e., a combined mechanism). The present study compares the SCC behaviour of the extruded Mg alloy RS66 and the traditional AZ91D alloy. The study also presents SCC tests under the imposed electrochemical conditions for the purpose of the comparison of the electrochemical mechanisms of SCC of the two alloys.

## 2. Experimental Procedure

### 2.1. Test Materials and Test Environment

**Figure 1:**
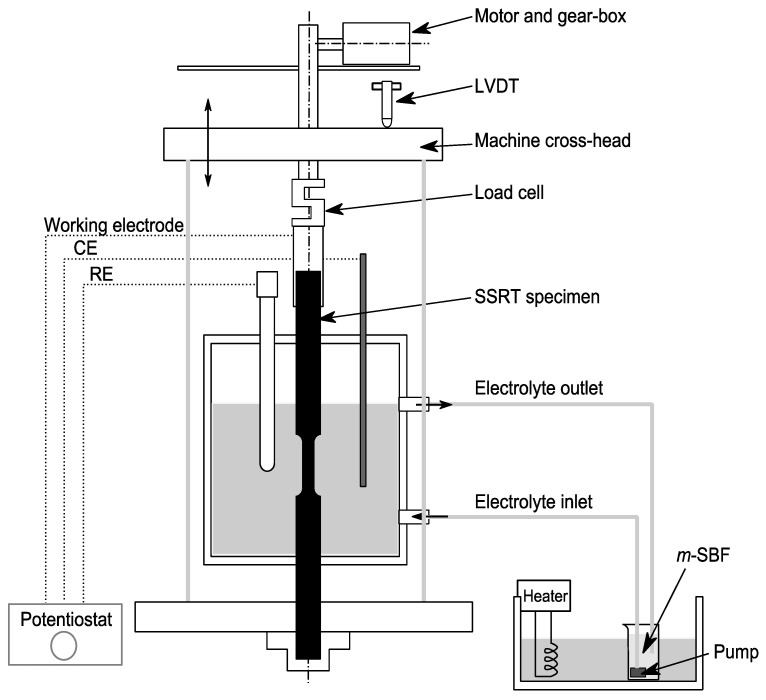
Schematic describing SSRT test set-up (RE: saturated calomel reference electrode and CE: platinum counter electrode) [16].

RS66 alloy (Mg-6.0%Zn-1.0%Y-0.6%Ce-0.6%Zr) tested in this study was received in the form of extruded rods. AZ91D alloy (as-cast) was used for comparative study. The test environment was a modified-simulated body fluid (*m*-SBF), whose composition is in Table 1. The *m*-SBF was circulated (at a flow rate of 15 mL/s) at 37 °C around the test specimen during the entire test duration, using a hot bath and a pump in the experimental set-up, as shown in Figure 1. The *m*-SBF solution was buffered with 2-(4-(2-hydroxyethyl)-1-piperazinyl) ethansulfonic acid (HEPES) for the purpose of maintaining the physiological pH of 7.4.

### 2.2. SCC Assessment by SSRT

Slow strain rate tensile (SSRT) testing setup (Figure 1) was employed for characterisation of the susceptibility of the test alloys to SCC. Dog-bone-type cylindrical specimens (gauge diameter of 3 mm and gauge length of 20 mm) were subjected to grinding with SiC paper up to a 2500 grit finish, followed by cleaning with acetone and deionised water. On the basis of the reported SSRT tests for characterisation of SCC of Mg alloys in chloride media [16,27], that employed strain rates in the range of 10^−7^ s^−1^, the specimens were subjected to tensile pulling to fracture at a strain rate of 3.1 × 10^−7^ s^−1^, while a linear variable displacement transducer (LVDT) was used for determination of elongation.

In order to electrochemically investigate the role of hydrogen, if any, in the mechanism of SCC of the two alloys, SSRT tests were carried out in *m*-SBF with the specimen held simultaneously and continuously at an imposed cathodic potential, i.e., −200 mV (vs. SCE) with respect to the open circuit potential (OCP). The regime of cathodic potential was identified through the potentiodynamic polarisation (PDP) tests, employing a potentiostat and a three-electrode cell (where the duly polished alloy coupon was the working electrode, a saturated calomel electrode (SCE) was the reference electrode, and a Pt-mesh was the counter electrode). For SSRT tests under the imposed cathodic potential, the SSRT specimens were held at a cathodic potential in order to suppress anodic dissolution, and to investigate whether the susceptibility to environment-assisted cracking enhanced under the condition of excessive hydrogen generation due to the cathodic changing, and little anodic dissolution. For this purpose, as seen in Figure 1, the SSRT test set-up was equipped with the potentiostat and a three-electrode cell (described earlier).

Reproducibility of SSRT and electrochemical corrosion data was examined by performing duplicate tests.

### 2.3. Fractography

A scanning electron microscope (SEM) was employed for fractography of the specimens that failed upon SSRT tests, to examine for the presence of SCC features (i.e., intergranular or transgranular mode of cracking). The fracture surfaces were cleaned with an inhibited cleaning medium of 10 wt.% AgNO_3_ + 20 wt.% CrO_3_ for 10 s. The inhibited cleaning medium was employed for avoiding undesirable dissolution of the fracture surface while achieving the required cleaning. After exposure to the cleaning solution, the samples were subjected to ultrasonic cleaning for 10 min in acetone and drying before SEM fractography.

## 3. Results and Discussion

### SCC Assessment by SSRT

**Figure 2:**
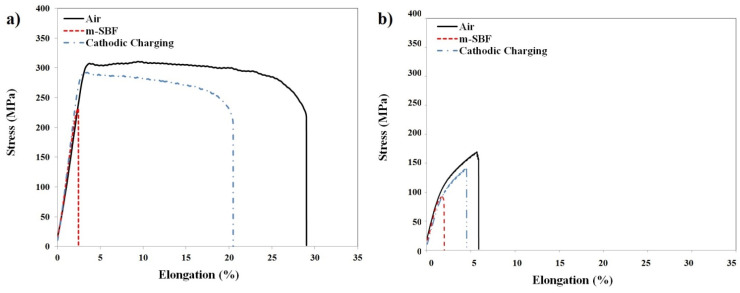
Stress vs. elongation plots of the two magnesium alloys subjected to SSRT (strain rate of 3.1 × 10^−7^ s^−1^) in air and *m*-SBF (with and without imposed cathodic potential): (**a**) RS66 and (**b**) AZ91D (note, the specimen tested in *m*-SBF without imposed potential represents open circuit potential, OCP).

Figure 2a and Figure 2b, respectively, present the stress vs. elongation plots for the RS66 and AZ91D alloys during SSRT (strain rate: 3.1 × 10^−7^ s^−1^) in air and *m*-SBF, as well as with the specimens under imposed cathodic potentials while immersed in *m*-SBF. Table 2 presents the comparison of the mechanical properties of the two alloys tested in *m*-SBF and air. The much superior strength of RS66 (than that of AZ91D) is attributed to its fine grain size [38], whereas its concurrent superior ductility is also attributed to the reported role of grain refinement in promoting the non-basal dislocation modes of magnesium alloys [40]. RS66 has nanometre-size strengthening precipitates (~80 nm) uniformly dispersed within the micron-size grains (~1.2 μm) of the matrix, as reported by earlier work on the same alloy by author Shechtman [38]. On the other hand, the grain size of the AZ91D alloy is much larger, as reported by earlier work on this alloy by author Singh Raman [13], which contributed to the alloy’s much inferior strength (besides the usually low ductility of as-cast alloys).

Both the alloys show considerably reduced ultimate tensile strength (UTS) and elongations-to-failure when tested in *m*-SBF solution (Figure 2 and Table 2), indicating each alloy to have possibly suffered embrittlement in *m*-SBF. However, in spite of its much superior mechanical properties in air, RS66 suffered much greater loss in elongation-to-failure, i.e., it had a greater susceptibility to SCC (as elaborated later).

**Figure 3:**
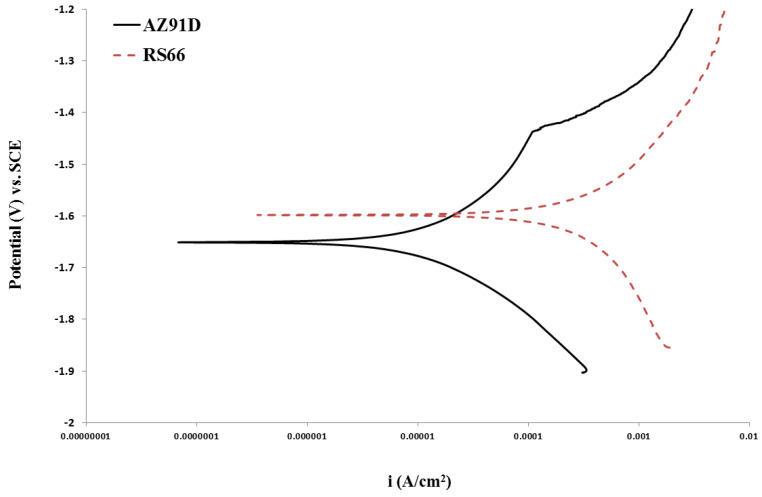
Potentiodynamic polarization plot for RS66 and AZ91D alloys in *m*-SBF at 37 °C.

Mg alloys suffer rapid corrosion in aqueous chloride solutions, which includes SBF, and, unlike other metals, Mg corrosion concurrently generates considerable hydrogen (even under anodic conditions due to the negative difference effect [41]). Mg alloys are also known to readily suffer localized corrosion/pitting [12,13]. Therefore, the loss in UTS and elongation-to-failure during the SSRT tests (without any imposed cathodic potential) in *m*-SBF (Table 2 and Figure 2) can either be a result of just the corrosion-assisted loss of the load-bearing cross-sectional area of the specimen gauge due to general and localized corrosion (which is not SCC) or it can be due to the simultaneous effect of corrosion and stress (that is SCC). To ascertain, the two alloys were subjected to a few critical SSRT tests under simultaneously and continuously imposed cathodic potentials, i.e., a condition under which hydrogen is expected to be the primary cause of embrittlement and dissolution will have a minimal effect. The PDP of the two alloys, as shown in Figure 3, determined the required information on their cathodic potential regimes in *m*-SBF, for carrying out SSRT tests at cathodic potentials. The PDP tests also generated corrosion current density (i_corr_) and corrosion potential (E_corr_) data.

The comparison of the SSRT plots generated in air, *m*-SBF, and with imposed cathodic potential in *m*-SBF (Figure 2) provides mechanistic clues. First, the elongation-to-failure for each alloy under imposed cathodic potential is considerably less than the corresponding elongation in air. This observation confirms that the hydrogen-assisted embrittlement contributed to the loss of ductility/elongation. This loss cannot be entirely attributed to pitting/corrosion-assisted loss of load-bearing area since the alloy is expected to suffer minimal anodic dissolution and pitting under an imposed cathodic potential. A more important observation for each alloy was that the specimen tested in the *m*-SBF at the open circuit potential had the least elongation-to-failure, as seen in Figure 2. The elongation-to-failure is considerably greater for the specimen tested in hydrogen-abundant conditions (the cathodic charging conditions), which suggests that hydrogen is not the exclusive reason for cracking. This inference also points towards the combined effect of anodic dissolution and hydrogen-assisted embrittlement, which is similar to the reported combined mechanism in the case of SCC of AZ91D in an aqueous chloride environment [16].

**Figure 4:**
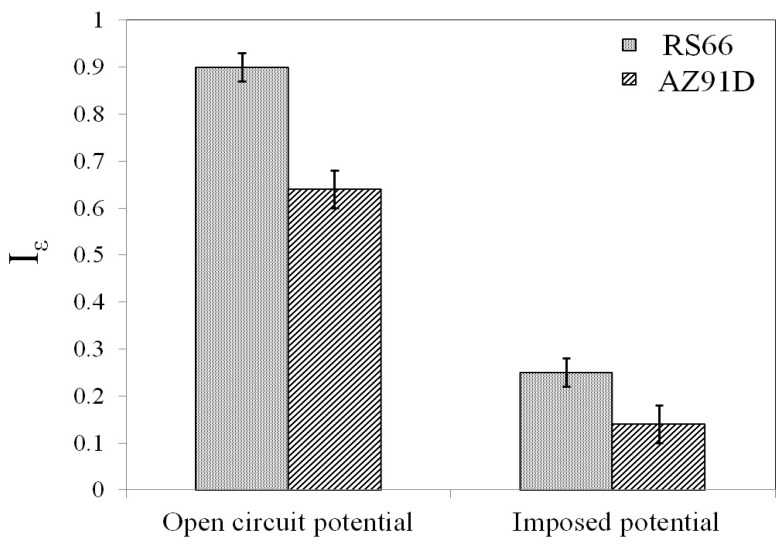
SCC susceptibility index of the two alloys during SSRT at OCP and imposed cathodic potential.

A closer scrutiny of the elongation-to-failure data for the two alloys under the cathodic charging and open circuit conditions (Figure 2) suggests the contribution of anodic dissolution to be considerably greater in the case of RS66 alloy, which is quantitatively represented through SCC susceptibility index data for open circuit and cathodic charging conditions (Figure 4). Loss in ductility/elongation (Ɛ) is one of the ways to define the SCC susceptibility index ($I_{Ɛ}$) [42]:

$$I_{Ɛ}=\frac{\left(Ɛ\;in\;air\right)-(Ɛ\;in\;corrosive\;environment\;at\;OCP\;or\;at\;imposed\;potential)}{Ɛ\;in\;air}$$

The susceptibility to SCC increases with the increase in $I_{Ɛ}$.

The lower susceptibility of the AZ91D alloy to SCC at OCP (i.e., lower $I_{Ɛ}$) is attributed to the Al content of the alloy that confers the ability to possess some passivation. The alloy’s tendency to passivate is indicated in Figure 3 by the sudden increase in current density at a potential around −1.43V, which is a characteristic of disruption in a passive film under the influence of anodic over-potential. The somewhat lower susceptibility of AZ91D alloy to environment-assisted embrittlement/fracture (i.e., lower $I_{Ɛ}$) at the imposed cathodic potential can be attributed to the alloy’s lower susceptibility to generate hydrogen at such potentials (as is evident from lower cathodic current densities for AZ91D alloy than those for RS66 over the entire cathodic range of the PDP plots in Figure 3), and hence, there was a lesser embrittling effect for AZ91D due to hydrogen.

## 4. Fractography

**Figure 5:**
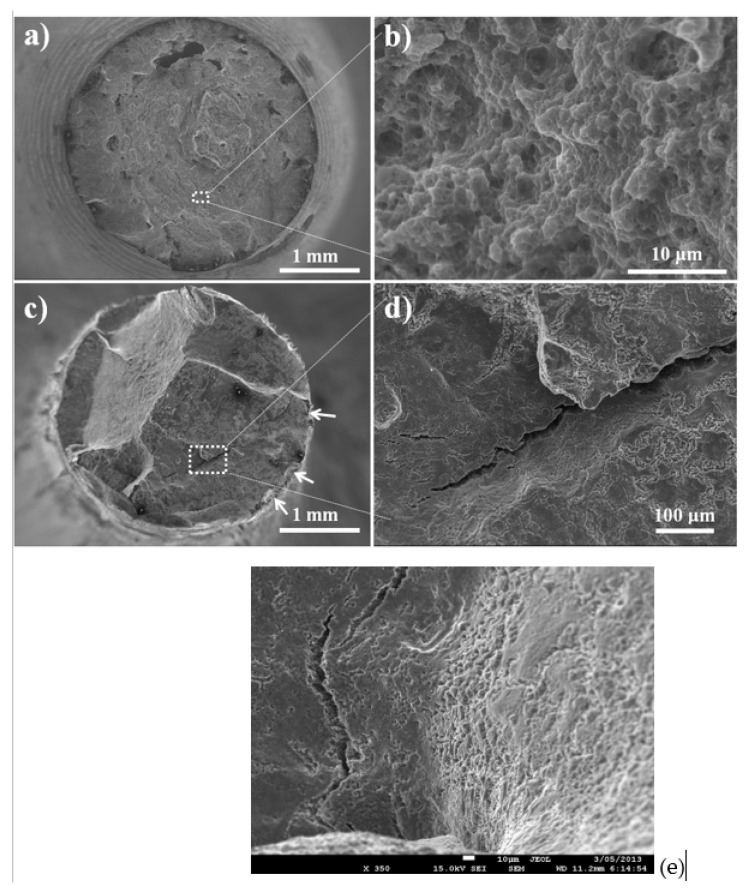
SEM fractographs of RS66 fractured upon SSRT: (**a**) overall fracture surface of a sample tested in air, (**b**) dimple formation on sample tested in air, (**c**) overall fracture surface of sample tested in *m*-SBF (arrows show pitting on circumference of the tested specimen), (**d**) evidence of brittle crack propagation of sample tested in *m*-SBF, and (**e**) area of transition from brittle cracking to overload ductile failure, and secondary cracks in the area of brittle cracking of sample tested in *m*-SBF.

**Figure 6:**
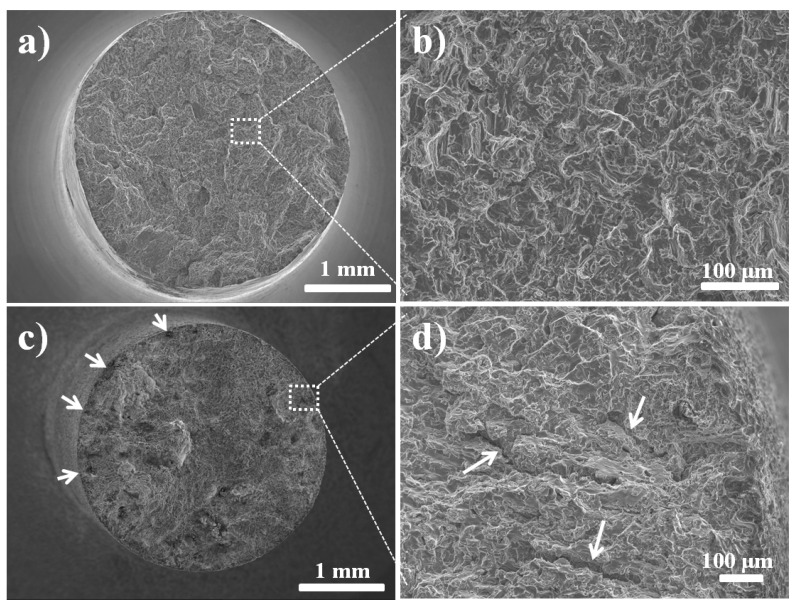
SEM fractographs of AZ91D fractured upon SSRT: (**a**) overall fracture surface of sample tested in air, (**b**) dimple formation on most parts of the fracture surface of the sample tested in air, (**c**) overall fracture surface of sample tested in *m*-SBF (arrows show pitting on circumference of the tested specimen), and (**d**) evidence of SCC, i.e., secondary cracking of sample tested in *m*-SBF.

SEM was employed for fractographic examination to confirm the SCC that was indicated by SSRT. Fractographs of the two alloys (Figure 5 and Figure 6) tested in air show dimples or quasi-cleavage formation at higher magnifications, over the entire fracture surface (Figure 5b and Figure 6b).

The two alloys tested in the *m*-SBF possess evidence of pitting at the sample circumferences (Figure 5c and Figure 6c), whereas considerable fractions of their fracture surface have features of embrittlement (Figure 5d and Figure 6d). The fracture surfaces in Figure 5d and Figure 6d also show the secondary cracking that is one of the distinctive features of SCC. The embrittlement feature of AZ91D has discernible facets (Figure 6d), which is consistent with those reported earlier for this alloy [16]. The embrittlement areas of the fracture surface of RS66 are less distinct (Figure 5d). This could possibly be attributed to the much finer grain size of the alloy [37]. Figure 5e shows an area of transition from such brittle cracking to mechanical overload failure (as evident from the dimples).

The SBF-assisted cracking of the two alloys is attributed to the combined mechanism; however, the two alloys showed different degrees of contributions from anodic dissolution and the hydrogen effect. The environment-assisted cracking of magnesium alloys in aqueous chloride is reported to progress via the localised mechanical or electrochemical disruption of protective corrosion films (such as that at the tip of an existing crack/discontinuity/defect), which creates a narrow opening for hydrogen entry into the alloy matrix, causing embrittlement that eventually leads to a premature fracture [14,43]. One of the earliest studies on the topic by Stampella et al. [43] suggested pits to be the primary cause of stress corrosion cracking of pure Mg in Na_2_SO_4_ medium, and a recent study on chloride-SCC of a Mg-Mn alloy [44] also reported pits to be the primary initiation sites for SCC, whereas hydrogen entry through the narrow opening contributed to embrittlement and crack propagation. The lesser ability of RS66 to passivate (as discussed earlier) is also manifest in considerably greater locations of localized attacks at the circumference of the RS66 sample (Figure 5c and Figure 6c), which is consistent with the greater loss of ductility of this alloy (as also discussed earlier).

The reason for choosing as-cast AZ91D alloy for comparison of SCC susceptibility of the extruded RS66 has been briefly described at the outset. As seen in Figure 4, the SCC susceptibility of the as-cast AZ91D was found to be lower (IƐ: ~0.65) than that of RS66 (IƐ: ~0.9), which has been attributed in earlier discussion to the high Al-content of the former. In this context, it is useful to note the high SCC susceptibility (IƐ: ~0.9) of the extruded version of another Al-free alloy (ZX10) [20].

Regarding future opportunities in assessing SBF-assisted cracking of magnesium alloys, it may also be necessary to simulate the corrosion under actual in-vivo conditions. The presence of organic constituents, such as proteins, glucose, fibrinogen, lipids, etc., in the actual in vivo body fluid, in addition to the inorganic constituents (that the commonly used SBF simulation for in vitro tests have) have been reported to considerably alter the nature of the surface film of magnesium alloys and influence their corrosion characteristics [4,45,46]. In fact, the addition of a protein to the Hanks’ solution has recently been demonstrated to alter the susceptibility to SCC of a magnesium alloy [22]. Therefore, there is a need of further investigation of SCC of RS66 alloy in more accurately simulated body fluids, considering the influence of the organic constituents such as protein and glucose, which is an unexplored field of immense social as well as technological importance.

## 5. Conclusions

In this study, resistance of a recently developed biocompatible and biodegradable Al-free extruded magnesium alloy, RS66, to stress corrosion cracking (SCC) in *m*-SBF was evaluated using slow strain rate testing (SSRT) and fractography. SCC resistance of this alloy was compared with that of a traditional Al-containing alloy, AZ91D. When tested in air, the RS66 alloy possessed considerably superior mechanical properties, as a result of its fine grain microstructure. Considerably lower elongations-to-failure in *m*-SBF as compared to those in air for both the alloys suggested their significant loss of ductility due to the corrosive environment. However, the elongations-to-failure in *m*-SBF were greater for RS66 alloy. The susceptibility of RS66 alloy to SCC was ~25% greater than that of AZ91D, which is attributed to the greater resistance of AZ91D to corrosion/localised corrosion because of its Al content. The SCC mechanism was the combined effect of hydrogen-assisted embrittlement and anodic dissolution.

## Figures and Tables

**Table 1:** Composition of reagents in 1000 mL of the *m*-SBF solution.

Reagents	Amount
NaCl	5.403 g
NaHCO_3_	0.504 g
Na_2_CO_3_	0.426 g
KCl	0.225 g
K_2_HPO_4_.3H_2_O	0.23 g
MgCl_2_.6H_2_O	0.311 g
0.2 mol L^−1^ NaOH	100 mL
HEPES	17.892 g
CaCl_2_	0.293 g
Na_2_SO_4_	0.072 g
1 mol L^−1^ NaOH	15 mL

**Table 2:** Mechanical properties of RS66 and AZ91D alloys subjected to SSRT (strain rate of 3.1 × 10^−7^ s^−1^) in air and *m*-SBF.

Alloy	In Air		In *m*-SBF	
	UTS (MPa)	Elongation to Failure (%)	UTS (MPa)	Elongation to Failure (%)
RS66	305.8 ± 4.6	27.2 ± 1.8	248.4 ± 17.2	2.7 ± 0.2
AZ91D	165.5 ± 4.5	5.4 ± 0.4	83.2 ± 8.7	1.9 ± 0.1

## Data Availability

Data are contained within the article.
